# Infant weight gain and motor development in relation to childhood adiposity and physical activity

**DOI:** 10.1016/j.jsampl.2025.100123

**Published:** 2025-11-28

**Authors:** Tomoko Aoyama, Yuki Hikihara, Masashi Watanabe, Hitoshi Wakabayashi, Hidemi Takimoto, Shigeho Tanaka

**Affiliations:** a Department of Social Medicine, National Center for Child Health and Development, 2-10-1 Okura, Setagaya-ku, Tokyo, 157-8535, Japan; b Liggins Institute, University of Auckland, 85 Park Road, Grafton, Auckland, 1023, New Zealand; c Faculty of Creative Engineering, Center for Liberal Arts, Chiba Institute of Technology, 2-1-1 Shibazono, Narashino-shi, Chiba, 275-0023, Japan; d Faculty of Education, Ibaraki University, 2-1-1 Bunkyo, Mito-shi, Ibaraki, 310-8512, Japan; e Faculty of Engineering, Hokkaido University, Kita 13, Nishi 8, Kita-ku, Sapporo-shi, Hokkaido, 060-8628, Japan; f National Institute of Health and Nutrition, National Institutes of Biomedical Innovation, Health and Nutrition, 3-17 Senrioka Shinmachi, Settsu-shi, Osaka, 566-0002, Japan; g Faculty of Nutrition, Kagawa Nutrition University, 3-9-21 Chiyoda, Sakado-shi, Saitama, 350-0288, Japan

**Keywords:** Birth weight, Child development, Motor skills, Obesity, Rapid weight gain

## Abstract

**Objectives:**

The long-term effects of infant weight gain and motor development on adiposity and physical activity remain unclear. This study investigates how these factors predict objectively assessed adiposity and physical activity at early school age, and whether these associations persist into preadolescence.

**Methods:**

This retrospective study with prospective follow-up included 223 first-grade children (aged 6–7) for initial assessments, including body fat percentage determined by dual-energy X-ray absorptiometry and moderate-to-vigorous physical activity (MVPA) assessed using an accelerometer. A four-year follow-up involved 216 fifth-grade children (aged 10–11). Data on weights measured at birth and at 1-, 3–4-, and 18-month checkups, along with ages at which six gross motor milestones were achieved, were extracted from the Maternal and Child Health Handbook.

**Results:**

Multivariable regression analyses, adjusted for sex, gestational age, height, school location, maternal age, and pre-pregnancy body mass index showed that rapid weight gain from birth to 18 months (*p* ​= ​0.01) and later age at standing with support (*p* ​< ​0.001) were independently associated with higher body fat assessed in the first grade, with a significant negative interaction (*p* ​= ​0.02). Later age at standing with support was also significantly associated with less MVPA time in the first grade (*p* ​= ​0.02). Among these, only the association between age at standing with support and body fat remained significant in the fifth grade (*p* ​< ​0.01).

**Conclusions:**

This study highlights the long-term implications of later achievement of motor milestones for future adiposity, persisting into preadolescence. Infant motor development can be an important determinant of future health.

## 1 Introduction

The foundational role of early-life growth—both pre- and postnatal—in shaping lifelong health has been increasingly recognized. A growing body of evidence links low birth weight and rapid weight gain during infancy to various adverse long-term health outcomes, including obesity [1,2] and physical inactivity [3]. Both adiposity [4,5] and physical activity [6,7] are key determinants of children’s current and future health. Thus, understanding how early-life weight gain impacts adiposity and physical activity during childhood is critical for developing effective interventions and parenting strategies to support lifelong health.

In addition to weight gain, recent studies indicate the significance of motor development during infancy in shaping lifelong health. Later achievement of gross motor milestones, even within normal developmental ranges, has been associated with increased adiposity and lower levels of physical activity in later life. For instance, achieving milestones such as crawling, standing with support, and walking with support at later ages predicts higher body fat in children aged 6–7 years [8]. Similarly, achieving independent walking at a later age predicts lower levels of objectively measured physical activity in children aged 4–7 years [9] and 6–12 years [10]. These findings highlight infant gross motor development as an important factor shaping long-term physical health.

Despite this growing evidence, weight gain and motor development are often discussed separately, leaving their relative importance in the context of lifelong health insufficiently understood. Moreover, the relationship between rapid weight gain during infancy and physical activity in childhood remains largely unexplored. A better understanding of how early-life factors collectively shape childhood adiposity and physical activity is essential for advancing a life-course approach to health.

Our previous study retrospectively investigated the associations between early-life factors, such as birth weight and motor development, and adiposity and objectively measured physical activity in early school-aged children (6–7 years) [11]. The results indicated that later achievement of certain motor milestones, particularly standing with support (age at which the child first stood), is associated with higher body fat and lower physical activity levels in childhood. However, that study did not account for weight gain during infancy, a potential predictor of childhood adiposity. The updated dataset for this study includes additional variables related to weight gain during infancy, as well as data from a four-year follow-up assessment in late elementary school [12]. This provides a unique opportunity to explore how early-life factors such as weight gain and motor development impact adiposity and physical activity across elementary school years. Therefore, this study aims to elucidate the associations of early-life weight gain and motor development (specifically age at achieving standing with support) with adiposity and physical activity at early school age. We also examined whether these relationships persist into late elementary school age.

## 2 Methods

### 2.1 Overview

This study involved a four-year prospective observation of 248 first-grade children (153 boys) recruited from six elementary schools in the Kanto region of Japan in 2012 and 2013. The study data included information on maternal health during pregnancy and child health from birth to the age of three years, collected from the Maternal and Child Health Handbook (MCHH) records (described below). Previously, we reported on the relationships of the ages at achieving six particular motor milestones (based on MCHH records) with adiposity indicated by the percentage of body fat [8] and with objectively measured physical activity [11] assessed at baseline. The present study expanded the dataset to include these same outcomes assessed at a four-year follow-up in the fifth grade in 2016 and 2017 [12]. This study was approved by the ethics committees of the National Institute of Health and Nutrition in Japan and Chiba Institute of Technology. Written explanations of the study procedures were provided to all participants and their parents, and written informed consent was obtained from the parents prior to participation.

### 2.2 Maternal and Child Health Handbook (MCHH)

The MCHH is a home-based health record issued by local governments when a woman registers her pregnancy in Japan [13]. It contains records of pregnancy checkups, delivery, and health checkups and immunizations for children from birth to school age, all provided by health professionals. Additionally, it includes sections for pregnant women to report pre-pregnancy conditions, and for parents to record their children’s development, including gross motor development. The pre-pregnancy body mass index (BMI, kg/m^2^) was calculated based on self-reported height and weight data. The methods employed for collecting these records were described in detail in our previous article [8].

### 2.3 Weight gain

Data on weight and length or height at birth and at 1, 3–4, and 18 months were extracted from the health checkup records in the MCHH. Rapid weight gain in infancy is often defined as a change in the weight-for-age standard deviation (SD) score of more than 0.67 SDs from birth to two years of age [1]. As the exact period of rapid weight gain during infancy predictive of future obesity is still under investigation, weight gain from birth to 1, 3–4, and 18 months of age was examined in this study. Weight-for-age SD scores were calculated as ([individual weight − standard mean weight]/standard weight SD), using a Microsoft Excel sheet (taikakubirthlongcross_v1.1.xlsx [accessed December 4, 2023]) developed and distributed by the Japanese Society for Pediatric Endocrinology (http://jspe.umin.jp/eng/index.html) and the Japanese Association for Human Auxology (https://auxology.jp/[in Japanese]). Changes in weight-for-age SD scores were analyzed as continuous variables, in accordance with a previous study [14].

### 2.4 Motor development

Information regarding gross motor development was sourced from parental records in the MCHH. The ages (in months) at which the following six gross motor developmental milestones were achieved were calculated: holding the head up, sitting, crawling, standing with support, walking with support, and independent walking [8,11]. Unlike height and weight measured by healthcare professionals during health checkups, motor development is voluntarily recorded by parents prior to each health checkup. Consequently, the motor development records were partially incomplete.

### 2.5 Anthropometry and body composition measurements in childhood

Weight, height, and body composition were assessed during the summer holidays in July/August in the first and fifth grades at the authors’ research institutions. The participants wore light clothing and no shoes when the height and weight measurements were taken. BMI was calculated in kg/m^2^ based on height and weight. Relative weight (%) was calculated as [individual weight (kg) − standard weight (kg)] / standard weight (kg) × 100. Standard weight (kg) was derived from the equation a × measured height (cm) – b (a and b are sex- and age-specific values, respectively) [15]. Participants were then classified into three categories: obese (≥ 20 %), normal (−20 to 20 %), or thin (≤ −20 %). Body composition was assessed using dual-energy X-ray absorptiometry (DXA) with a QDR-4500 densitometer (Hologic, Inc., Waltham, MA, USA) equipped with Hologic QDR software, version 12.7.3. All scans were performed with sex and age set according to each participant’s chronological age and biological sex. The same software version was used throughout the study period (2012–2017) to maintain comparability of body composition estimates over time. While the algorithm used by the software to estimate body fat is proprietary, this consistent use of the same version helps minimize potential variation. The body fat–body weight ratio was determined as the percentage of body fat.

### 2.6 Physical activity

For physical activity measurements, participants wore a tri-axial accelerometer (Active style Pro HJA-350IT, Omron Healthcare, Kyoto, Japan) on the lower back for 14 days during the school term in October/November in both the first and fifth grades. The device samples acceleration at 32 Hz with a measurement range of ±6 G. We used 10-s epochs for data recording. The Omron Active style Pro is considered one of the most accurate wearable devices among the 12 tested, showing a significant positive correlation with physical activity energy expenditure measured by the doubly labelled water method and providing comparable estimates in adults [16]. This high accuracy is partly due to its proprietary algorithm, which distinguishes between locomotor activities (e.g., walking, running) and non-locomotor activities (e.g., household chores) based on changes in body posture [17].

This algorithm works by calculating the ratio of unfiltered synthetic acceleration to filtered synthetic acceleration. In a validation study involving children aged 6–12 years, using an unfiltered/filtered synthetic acceleration ratio threshold achieved an average correct classification rate of approximately 99 %, demonstrating high accuracy in classifying activity types in a pediatric population [18]. Using this discrimination method, physical activity data for elementary school children were converted with the following equations:

Locomotor activities: 0.6237 × metabolic equivalents value + 0.2411.

Non-locomotor activities: 0.6145 × metabolic equivalents value + 0.5573.

The specific protocol employed in this study has been outlined in previous publications [11,12,19]. We analyzed data collected between 7:00 AM and 9:00 PM, excluding any data recorded while the children were sleeping. Periods of consecutive zero counts lasting less than 20 min, which indicate no signal, were included as valid accelerometer wear time. Children with wear times of 10 h or more on at least 5 weekdays and 2 weekend days were included in the analysis to meet the minimum criterion of 4–5 valid days for school-aged children [20]. The mean daily time spent in moderate-to-vigorous physical activity (MVPA), defined as ≥ 3.0 metabolic equivalents, was calculated by weighting the data for 5 weekdays and 2 weekend days as follows:

weighted average = ([mean for weekdays × 5] + [mean for weekend days × 2])/7.

In the analysis including physical activity data, we excluded 14 participants in the first grade and 29 participants in the fifth grade due to accelerometer data not meeting the inclusion criteria. A macro program developed and distributed by the Japan Physical Activity Research Platform (http://paplatform.umin.jp [in Japanese]) was used for accelerometer data processing.

### 2.7 Statistical analysis

Participants’ physical characteristics were summarized using means and SDs. Changes in outcomes from the first to the fifth grades were tested using paired t-tests. Partial correlations among early-life factors, including weight gain and ages at achieving motor milestones, were calculated with adjustment for sex and gestational age (Supplementary Table 1).

Adjusted univariate analyses were first conducted to screen variables, followed by multivariable regression analyses to evaluate the joint associations of weight gain and motor development with later health indicators. For each outcome—body fat percentages and physical activity assessed in the first and fifth grades—models were adjusted sequentially: Model 1 for sex and gestational age; Model 2 additionally for height at outcome assessment and school location (prefecture); and Model 3 further for maternal pre-pregnancy BMI and maternal age at delivery. Models predicting physical activity were additionally adjusted for accelerometer wear time (min/day). Results are presented as unstandardized regression coefficients with 95 % confidence intervals (CIs).

**Fig. 1:**
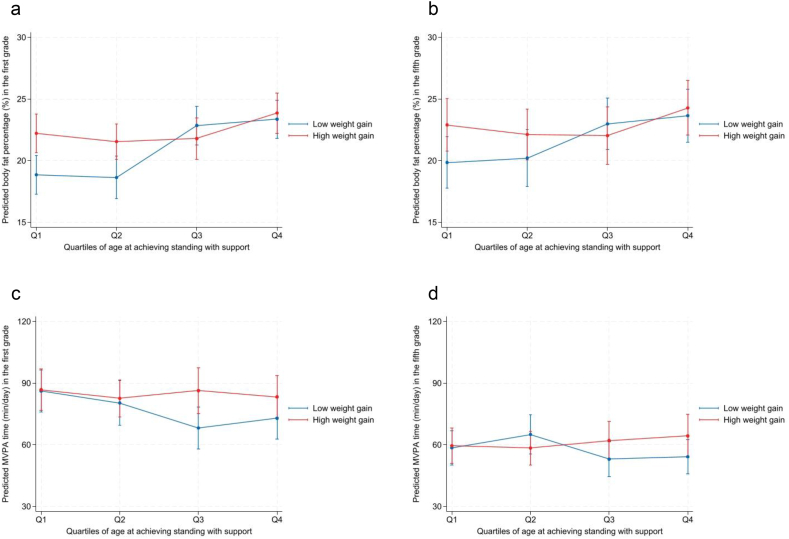
**Adiposity and physical activityin thefirstand fifth gradesby quartiles ofage at achieving standing with support.** Predicted body fat percentages (a, b) and MVPA time (c, d) with 95 % confidence intervals are shown according to quartiles of age at achieving standing with support and median split of weight gain from birth to 18 months. Estimates are based on multivariable regression models adjusting for sex, gestational age, height at outcome assessment, school location, maternal pre-pregnancy body mass index, maternal age at delivery, and accelerometer wear time in models with MVPA as the outcome; the interaction between weight gain and motor milestone achievement was included in the model. Q1 (< 7.4 months), Q2 (7.4–8.1 months), Q3 (8.2–9.0 months), and Q4 (> 9.0 months).

In the multivariable models, weight gain from birth to 18 months and age at achieving standing with support were included simultaneously, and their interaction term was tested. For illustrative purposes, age at achieving standing with support was categorized into quartiles and weight gain was dichotomized at the median. Predicted body fat percentages and physical activity for the first and fifth grades were calculated for each combination of standing-age quartile and weight-gain category using models adjusted for all covariates. These estimates, along with 95 % CIs, were plotted to visualize the interaction (Fig. 1).

Statistical analyses were performed using Stata, version 18.0 (StataCorp LLC, College Station, TX, USA). The statistical significance level was set at *p* < 0.05.

## 3 Results

Our analysis focused on a cohort of 223 children (136 boys) in the first grade, after excluding 1 child born with an extremely low birth weight (< 1.5 kg), 14 children without any of the 6 motor development records, 2 children without records of maternal pre-pregnancy BMI, and 8 children for whom MCHH records were unavailable. While there were 7 dropouts between the first and fifth grades, 216 (132 boys) out of the 223 children attended the follow-up assessment in the fifth grade, indicating a notably high follow-up rate (97 %) in this analysis cohort.

Table 1 presents the participants’ characteristics. During the four-year follow-up, all the indicators for physical health changed significantly (*p* < 0.001), except for body fat percentage (paired t-test, *p* = 0.08). Height increased by 22.9 cm and weight increased by 12.9 kg, while MVPA decreased by 23.2 min/day. Based on relative weight, 1.8 % (*n* = 4) were categorized as obese in the first grade and 4.6 % (*n* = 10) in the fifth grade. None were categorized as thin in the first grade, whereas 3.7 % (*n* = 8) were categorized as thin in the fifth grade. Regarding the current guideline-based recommendations for engagement in MVPA for 60 min/day [6], 18.7 % (*n* = 39) and 63.1 % (*n* = 123) did not meet the guideline in the first and fifth grades, respectively.

**Table 1:** Participants' characteristics.

Variables		n	Mean (SD)	Range
**Early life**				
Maternal pre-pregnancy BMI (kg/m^2^)		223	20.3 (2.7)	16.1–35.4
Maternal age at delivery (years)		223	31.9 (4.6)	21–44
Gestational age at delivery (weeks)		223	38.9 (1.5)	32–41
Height/length	Birth (cm)	223	48.9 (2.1)	43.0–55.0
	1 month (cm)	218	53.2 (2.2)	46.7–58.0
	3–4 months (cm)	220	62.1 (2.5)	54.8–68.0
	18 months (cm)	220	80.6 (2.8)	73.5–88.0
Weight	Birth (g)	223	3009 (409)	1614–4064
	1 month (g)	223	4123 (546)	2544–5612
	3–4 months (g)	221	6631 (764)	4210–8845
	18 months (kg)	220	10.6 (1.0)	8.0–13.6
Weight gain (change in weight-for-age SD score)	Birth to 1 month	218	0.3 (1.3)	−3.5–3.6
	Birth to 3–4 months	220	0.2 (1.0)	−2.8–2.5
	Birth to 18 months	220	0.2 (1.0)	−2.3–3.1
Ages at achieving motor milestones (months)	Holding the head up	133	3.3 (0.6)	1.4–5.4
	Sitting	105	6.5 (0.8)	4.8–9.0
	Crawling	158	8.0 (1.5)	3.8–12.6
	Standing with support	154	8.3 (1.1)	6.2–12.1
	Walking with support	121	9.5 (1.5)	6.5–13.9
	Independent walking	186	12.8 (1.9)	9.0–19.0
**School age**				
First grade	Age (months)	223	82.9 (3.5)	76–89
	Height (cm)	223	119 (4.9)	107.1–132.1
	Weight (kg)	223	21.6 (3.0)	16.1–33.4
	BMI (kg/m^2^)	223	15.2 (1.4)	12.8–22.3
	Body fat percentage (%)	223	21.7 (4.5)	13.1–37.2
	MVPA (min/day)	209	81.1 (22.6)	33.5–168.8
Fifth grade	Age (months)	216	130.8 (3.5)	122–137
	Height (cm)	216	141.9 (6.1)	126.7–158.5
	Weight (kg)	216	34.5 (6.0)	24.2–58.3
	BMI (kg/m^2^)	216	17.0 (2.0)	13.2–24.7
	Body fat percentage (%)	214	22.1 (5.4)	12.8–43.3
	MVPA (min/day)	195	58.0 (20.6)	21.8–120.9

Abbreviations: BMI = body mass index; MVPA = moderate-to-vigorous physical activity; SD = standard deviation.

Partial correlation analysis adjusting for sex and gestational age showed weak to strong positive correlations among weight gain indicators (*r* = 0.26 to 0.63) and among ages at achieving motor milestones (*r* = 0.25 to 0.73). Weight gain during infancy was largely independent of ages at which motor milestones were achieved, except for weak negative correlations between weight gain from birth to 1 month (*r* = −0.17, *p* = 0.02) and from birth to 3–4 months (*r* = −0.16, *p* = 0.03) and age at achieving independent walking (see Supplementary Table 1).

The results of the univariate analysis with adjustment for covariates are presented in Table 2. No statistically significant associations were observed between birth weight (SD score) and either body fat percentage or physical activity assessed in the first and fifth grades. As an overall trend, weight gain up to 18 months showed consistent positive correlations with body fat percentage and MVPA measured in the first and fifth grades, with some exceptions. Among these, only the positive association between weight gain from birth to 18 months and MVPA in the first grade was significant (*B* = 3.64, *p* < 0.01), after adjustment for covariates.

**Table 2:** Univariable associations of infant weight gain and motor development with adiposity and physical activity at early and late elementary school.

	First grade			Fifth grade		
Body fat percentage (%)	Model 1	Model 2	Model 3	Model 1	Model 2	Model 3
Birth weight (SD score)	0.20 (−0.33 to 0.74)	−0.06 (−0.59 to 0.46)	−0.24 (−0.76 to 0.28)	−0.05 (−0.75 to 0.65)	−0.19 (−0.89 to 0.51)	−0.46 (−1.13 to 0.22)
***Weight gain (change in******weight-for-age******SDscore)***						
Birth to 1 mo	−0.21 (−0.65 to 0.22)	−0.04 (−0.46 to 0.39)	0.04 (−0.37 to 0.46)	−0.24 (−0.81 to 0.33)	−0.11 (−0.68 to 0.46)	0.04 (−0.51 to 0.59)
Birth to 3–4 mo	0.36 (−0.22 to 0.94)	0.35 (−0.20 to 0.91)	0.43 (−0.12 to 0.97)	0.46 (−0.30 to 1.23)	0.43 (−0.33 to 1.18)	0.53 (−0.19 to 1.25)
Birth to 18 mo	0.48 (−0.06 to 1.02)	0.32 (−0.21 to 0.85)	0.31 (−0.21 to 0.82)	0.27 (−0.43 to 0.97)	0.24 (−0.47 to 0.95)	0.20 (−0.47 to 0.87)
***Ages at achieving motormilestones(months)***						
Holding the head up	0.98 (−0.17 to 2.14)	1.06 (−0.07 to 2.18)	1.04 (−0.03 to 2.11)	0.3 (−1.21 to 1.8)	0.26 (−1.25 to 1.77)	0.30 (−1.12 to 1.72)
Sitting	0.35 (−0.59 to 1.30)	0.49 (−0.43 to 1.41)	0.53 (−0.41 to 1.47)	0.23 (−0.98 to 1.44)	0.37 (−0.79 to 1.52)	0.46 (−0.70 to 1.62)
Crawling	**0.73 (0.28 to 1.17)**∗∗	**0.64 (0.21 to 1.07)**∗∗	**0.76 (0.34 to 1.18)**∗∗∗	0.41 (−0.16 to 0.98)	0.36 (−0.2 to 0.92)	0.53 (−0.01 to 1.07)
Standing with support	**1.06 (0.50 to 1.62)**∗∗∗	**1.04 (0.50 to 1.58)**∗∗∗	**1.14 (0.63 to 1.65)**∗∗∗	**0.86 (0.14 to 1.57)**∗	**0.88 (0.18 to 1.59)**∗	**1.02 (0.35 to 1.69)**∗∗
Walking with support	**0.61 (0.11 to 1.11)**∗	**0.52 (0.02 to 1.01)**∗	**0.65 (0.18 to 1.13)**∗∗	0.61 (−0.05 to 1.27)	0.54 (−0.11 to 1.2)	**0.67 (0.06 to 1.28)**∗
Independent walking	0.04 (−0.29 to 0.37)	−0.06 (−0.38 to 0.25)	−0.02 (−0.33 to 0.30)	0.06 (−0.34 to 0.47)	0.00 (−0.4 to 0.4)	0.07 (−0.33 to 0.47)

**MVPA (min/day)**	**Model 1**	**Model 2**	**Model 3**	**Model 1**	**Model 2**	**Model 3**

Birth weight (SD score)	−1.82 (−4.60 to 0.95)	−2.08 (−4.91 to 0.76)	−2.27 (−5.15 to 0.60)	−0.42 (−2.77 to 1.93)	0.11 (−2.26 to 2.48)	0.15 (−2.27 to 2.57)
***Weight gain (change in******weight-for-age******SDscore)***						
Birth to 1 mo	**2.59 (0.41 to 4.76)**∗	**2.30 (0.12 to 4.48)**∗	2.10 (−0.12 to 4.31)	0.91 (−1.02 to 2.84)	0.30 (−1.63 to 2.24)	0.24 (−1.73 to 2.21)
Birth to 3–4 mo	1.39 (−1.67 to 4.44)	1.48 (−1.52 to 4.48)	1.30 (−1.71 to 4.31)	−0.34 (−2.88 to 2.2)	−0.02 (−2.54 to 2.49)	−0.09 (−2.64 to 2.46)
Birth to 18 mo	**3.60 (0.86 to 6.34)**∗	**3.59 (0.86 to 6.32)**∗	**3.64 (0.91 to 6.37)**∗∗	1.42 (−0.90 to 3.74)	2.09 (−0.24 to 4.42)	2.09 (−0.25 to 4.43)
***Ages at achieving motormilestones(months)***						
Holding the head up	−0.08 (−6.28 to 6.11)	−0.43 (−6.65 to 5.79)	−0.26 (−6.49 to 5.98)	−2.22 (−7.81 to 3.37)	−2.32 (−7.99 to 3.36)	−2.46 (−8.21 to 3.28)
Sitting	0.71 (−4.81 to 6.24)	0.64 (−4.61 to 5.89)	1.43 (−3.82 to 6.68)	−2.75 (−6.99 to 1.50)	−2.34 (−6.69 to 2.00)	−2.07 (−6.53 to 2.39)
Crawling	**−2.65 (−5.00 to−0.30)**∗	**−2.33 (−4.65 to−0.02)**∗	**−2.54 (−4.87 to−0.21)**∗	−0.67 (−2.78 to 1.44)	−0.39 (−2.50 to 1.72)	−0.44 (−2.60 to 1.72)
Standing with support	**−4.44 (−7.69 to−1.19)**∗∗	**−4.20 (−7.40 to−1.01)**∗	**−4.23 (−7.43 to−1.04)**∗	−0.92 (−3.77 to 1.94)	−0.85 (−3.68 to 1.98)	−0.88 (−3.74 to 1.98)
Walking with support	−2.61 (−5.41 to 0.18)	−1.99 (−4.81 to 0.83)	−1.98 (−4.90 to 0.94)	0.34 (−2.10 to 2.78)	0.35 (−2.13 to 2.82)	0.13 (−2.41 to 2.67)
Independent walking	−1.38 (−2.97 to 0.22)	−1.27 (−2.87 to 0.33)	−1.14 (−2.77 to 0.48)	0.22 (−1.23 to 1.67)	0.15 (−1.30 to 1.60)	0.11 (−1.37 to 1.59)

Values are unstandardized regression coefficients with 95 % confidence intervals.

Abbreviations: MVPA = moderate-to-vigorous physical activity; SD = standard deviation.

Model 1: Adjusted for sex and gestational age (and additionally for accelerometer wear time in models with MVPA as the outcome).

Model 2: Model 1 plus adjustment for height at outcome assessment and school location.

Model 3: Model 2 plus adjustment for maternal pre-pregnancy BMI and maternal age at delivery.

∗*p* < 0.05.

∗∗*p* < 0.01.

∗∗∗*p* < 0.001.

Ages at achieving motor milestones showed an overall tendency of positive correlations with body fat percentage and negative correlations with physical activity measured in the first and fifth grades, with a few exceptions. Of these, five associations reached statistical significance in the first grade after adjustment for covariates. Specifically, the ages at achieving three milestones were positively correlated with body fat percentage in the first grade (crawling: *B* = 0.76, *p* < 0.001; standing with support: *B* = 1.14, *p* < 0.001; walking with support: *B* = 0.65, *p* < 0.01). The ages at achieving two milestones were significantly and negatively correlated with MVPA measured in the first grade (crawling: *B* = −2.54, *p* < 0.05; standing with support: *B* = −4.23, *p* < 0.01). Of these five significant associations observed in the first grade, two remained significant in the fifth grade. In the fifth grade, the ages at achieving standing with support (*B* = 1.02, *p* < 0.01) and walking with support (*B* = 0.67, *p* < 0.05) were associated with body fat percentage.

Multivariable regression analysis results for adiposity and physical activity in the first and fifth grades are presented in Table 3. For adiposity, weight gain from birth to 18 months was positively associated with body fat percentage in the first grade (*B* = 5.58; 95 % CI, 1.23 to 9.93; *p* < 0.05), but this association was not statistically significant in the fifth grade (*B* = 4.78; 95 % CI, −0.97 to 10.53). Later age at achieving standing with support was significantly associated with higher body fat in both the first grade (*B* = 1.11; 95 % CI, 0.61 to 1.61; *p* < 0.001) and the fifth grade (*B* = 1.01; 95 % CI, 0.34 to 1.67; *p* < 0.01). An interaction effect between weight gain and standing with support was significant in the first grade (*B* = −0.63; 95 % CI, −1.17 to −0.10; *p* < 0.05), but not significant in the fifth grade (*B* = −0.54; 95 % CI, −1.25 to 0.17), as illustrated in Fig. 1.

**Table 3:** Multivariable models of infant weight gain and motor development in relation to adiposity and physical activity at early and late elementary school.

	First grade	Fifth grade
Body fat percentage (%)	*n* = 152	*n* = 147
Weight gain, birth to 18 mo	**5.58 (1.23 to 9.93)**∗	4.78 (−0.97 to 10.53)
Age at standing with support (mo)	**1.11 (0.61 to 1.61)**∗∗∗	**1.01 (0.34 to 1.67)**∗∗
Interaction (weight gain × standing with support)	**−0.63 (−1.17 to−0.10)**∗	−0.54 (−1.25 to 0.17)

**MVPA (min/day)**	***n*****= 135**	***n*****= 132**

Weight gain, birth to 18 mo	−21.23 (−49.69 to 7.23)	−6.01 (−29.55 to 17.52)
Age at standing with support (mo)	**−3.82 (−7.06 to−0.58)**∗	−0.97 (−3.86 to 1.93)
Interaction (weight gain × standing with support)	3.07 (−0.42 to 6.56)	1.06 (−1.85 to 3.96)

Values are unstandardized regression coefficients with 95 % confidence intervals.

Abbreviations: MVPA = moderate-to-vigorous physical activity.

All models were adjusted for sex, gestational age, height at outcome assessment, school location, maternal pre-pregnancy BMI, and maternal age at delivery (and additionally for accelerometer wear time in models with MVPA as the outcome).

∗*p* < 0.05.

∗∗*p* < 0.01.

∗∗∗*p* < 0.001.

Regarding MVPA, weight gain from birth to 18 months was negatively, but not significantly, associated with MVPA in both the first grade (*B* = −21.23; 95 % CI, −49.69 to 7.23) and the fifth grade (*B* = −6.01; 95 % CI, −29.55 to 17.52) (Table 3). Later age at achieving standing with support was significantly associated with lower MVPA in the first grade (*B* = −3.82; 95 % CI, −7.06 to −0.58; *p* < 0.05) but not in the fifth grade (*B* = −0.97; 95 % CI, −3.86 to 1.93). Interaction effects for MVPA were not statistically significant at either time point (Fig. 1).

## 4 Discussion

In this study, the ages at which gross motor milestones were achieved generally fell within the reported “windows of achievement” for healthy children [21], indicating that gross motor development in our cohort was essentially normal. Additionally, the prevalence of obesity in our study (1.8 % in the first grade; 4.6 % in the fifth grade) was lower than national averages for Japanese children (4.1 % at 6 years in 2013; 9.6 % at 10 years in 2017) [22], suggesting that our cohort represents a relatively healthy population with a narrower range of body composition.

Our analyses revealed that later achievement of standing with support was significantly associated with adiposity in the both first and fifth grades, independent of weight gain from birth to 18 months. To our knowledge, this study is the first to demonstrate a link between motor development during infancy and preadolescent adiposity measured by DXA. These findings suggest that motor development in infancy may be a key factor in the risk of preadolescent obesity, which substantially increases the likelihood of adult obesity. Supporting this, a meta-analysis found that children and adolescents with obesity were approximately five times more likely to become obese in adulthood compared to those who were not obese [5].

In contrast, greater weight gain from birth to 18 months was associated with higher body fat percentage in the first grade, consistent with a recent meta-analysis [1]. However, this association did not persist into the fifth grade, suggesting that the influence of early weight gain may diminish as children grow, possibly due to environmental or lifestyle factors. Interestingly, the effect of rapid weight gain from birth to 18 months was attenuated in children who achieved standing with support later, illustrating an interaction between early-life factors. This may reflect the timing of developmental events: standing with support typically develops around 7.4 months (1st–99th percentile: 4.8–11.4 months) [23], whereas the rapid weight gain measured in this study spanned birth to 18 months. Thus, the impact of an existing risk factor (later standing) can modify the effect of a subsequent risk factor (rapid weight gain) on adiposity at early school age. In other words, the impact of rapid weight gain on body composition is not uniform but depends on earlier-established developmental characteristics. Understanding these interactions is important for identifying children at highest risk and for designing timely interventions that consider both early growth patterns and motor development.

The association between later achievement of standing with support and lower MVPA observed in the first grade did not persist into the fifth grade, suggesting that infant motor development may have a limited influence on physical activity during school age. Environmental and social factors, such as participation in sports clubs or time spent in cram schools, may increasingly shape activity patterns in late elementary school.

Collectively, these findings highlight the importance of monitoring motor development milestones, such as standing, as key risk indicators for future obesity and physical inactivity. Although the influence of motor development on physical inactivity appears limited and does not persist beyond adolescence, its impact on adiposity seems to last at least until preadolescence. Early weight gain from birth to 18 months can also predict adiposity at early school age; however, since achievement of standing typically occurs earlier, it offers an opportunity for even earlier intervention. Caregivers and health providers should therefore focus not only on monitoring weight but also on supporting timely motor development to effectively prevent future obesity.

This study has several limitations. First, due to its design, the study sample may have been somewhat biased toward children who are physically confident and enjoy exercise [12]. Validation of these findings in population-based cohorts is needed. Second, as motor development data were derived from parental records, reporting bias cannot be ruled out. Although parental assessments of their children’s motor milestone achievements are fairly reliable [24], the mechanism for addressing missing data on motor development remains unclear. Third, sex-specific analyses were not performed because of the small sample size; further validation based on a larger and more representative sample is needed, including a thorough investigation of potential sex-based differences. Despite these limitations, the study’s strengths include a high follow-up rate (97 %) and the use of robust methods, such as assessing adiposity with DXA and measuring physical activity with a tri-axial accelerometer.

## 5 Conclusion

In summary, weight gain from birth to 18 months and age at achieving standing with support were each independently associated with adiposity and physical activity at early school age, but only motor development remained linked to adiposity at late elementary school age. These results suggest that infant motor development, rather than weight gain, may play a more critical and lasting role in obesity prevention during childhood. While early-life weight gain often receives primary attention as a risk factor for later obesity, our results demonstrate the importance of monitoring and supporting infant motor milestones such as standing, emphasizing their potential significance for lifelong health.

**Key points**

• Later achievement of standing with support was associated with higher body fat in both the first and fifth grades, and with lower MVPA in the first grade.
• Rapid weight gain from birth to 18 months was linked to higher body fat in the first grade and showed a significant negative interaction with the age at achieving standing with support.
• Most associations between early-life exposures and later health outcomes were attenuated by the fifth grade, except for the robust relationship between the age at achieving standing with support and body fat.

## Funding

This work was supported by a Grant-in-Aid for JSPS Fellows (JP13J07359 to TA) and Grants-in-Aid for Young Scientists (B) (JP24700759 to YH and JP25750373 to MW) from the Japan Society for the Promotion of Science. One of the authors (HW) was supported by the Yamaha Motor Foundation for Sports.

## Declaration of competing interest

The authors declare that they have no known competing financial interests or personal relationships that could have appeared to influence the work reported in this paper.
