# Supplementary material for: Infant weight gain and motor development in relation to childhood adiposity and physical activity
Source: JSAMS Plus. 2025 Nov 28;6:100123. doi: 10.1016/j.jsampl.2025.100123 (PMC13008444; doi:10.1016/j.jsampl.2025.100123)
Supplement: Multimedia component 1 [file mmc1.docx]

**Supplementary Table 1**

Partial correlations among early-life factors.

|  | | Maternal pre-pregnancy BMI | Maternal age at delivery | Birth weight (SD score) | **Weight gain** | | | **Ages at achieving motor milestones** | | | | |
| --- | --- | --- | --- | --- | --- | --- | --- | --- | --- | --- | --- | --- |
|  |  |  |  |  | Birth to 1 mo | Birth to 3–4 mo | Birth to 18 mo | Holding the head up | Sitting | Crawling | Standing with support | Walking with support |
| Maternal age at delivery | | **0.14^*^** |  |  |  |  |  |  |  |  |  |  |
| Birth weight (SD score) | | **0.17^*^** | −0.07 |  |  |  |  |  |  |  |  |  |
| **Weight gain** | Birth to 1 mo | **−0.14^*^** | **−0.18^**^** | **−0.37^***^** |  |  |  |  |  |  |  |  |
|  | Birth to 3–4 mo | −0.07 | −0.10 | **−0.63^***^** | **0.47^***^** |  |  |  |  |  |  |  |
|  | Birth to 18 mo | 0.03 | 0.01 | **−0.62^***^** | **0.26^***^** | **0.63^***^** |  |  |  |  |  |  |
| **Ages at achieving motor milestones** | Holding the head up | 0.05 | 0.01 | 0.00 | −0.14 | −0.02 | 0.09 |  |  |  |  |  |
|  | Sitting | −0.02 | 0.15 | −0.12 | 0.02 | −0.11 | 0.08 | **0.46^***^** |  |  |  |  |
|  | Crawling | −0.14 | −0.04 | 0.04 | −0.02 | −0.06 | 0.01 | **0.39^***^** | **0.27^*^** |  |  |  |
|  | Standing with support | −0.08 | 0.03 | 0.06 | 0.00 | −0.02 | 0.01 | **0.25^*^** | **0.33^**^** | **0.73^***^** |  |  |
|  | Walking with support | −0.08 | **0.22^*^** | 0.07 | −0.07 | −0.03 | 0.02 | **0.25^*^** | **0.36^**^** | **0.61^***^** | **0.70^***^** |  |
|  | Independent walking | −0.04 | 0.14 | 0.07 | **−0.17^*^** | **−0.16^*^** | −0.01 | **0.25^**^** | **0.36^***^** | **0.43^***^** | **0.48^***^** | **0.49^***^** |

Partial correlation coefficients among early-life factors, including weight gain (change in weight-for-age SD score) and ages (in months) at achieving six motor milestones in infancy, adjusted for sex and gestational age.

Abbreviations: BMI = body mass index; SD = standard deviation.

*^*^p* < 0.05.

*^**^p* < 0.01.

*^***^p* < 0.001.
